# Creatine supplementation during pregnancy: summary of experimental studies suggesting a treatment to improve fetal and neonatal morbidity and reduce mortality in high-risk human pregnancy

**DOI:** 10.1186/1471-2393-14-150

**Published:** 2014-04-27

**Authors:** Hayley Dickinson, Stacey Ellery, Zoe Ireland, Domenic LaRosa, Rodney Snow, David W Walker

**Affiliations:** 1 The Ritchie Centre, MIMR-PHI Institute of Medical Research, Monash University, 27-31 Wright St., Clayton, Melbourne 3168 Australia; 2 Clinical Research Centre, University of Queensland, Brisbane, Australia; 3 Centre for Physical Activity & Nutrition, Deakin University, Burwood, Melbourne, Australia; 4 Department of Obstetrics & Gynaecology, Monash Medical Centre, Clayton, Melbourne, Australia

## Abstract

While the use of creatine in human pregnancy is yet to be fully evaluated, its long-term use in healthy adults appears to be safe, and its well documented neuroprotective properties have recently been extended by demonstrations that creatine improves cognitive function in normal and elderly people, and motor skills in sleep-deprived subjects. Creatine has many actions likely to benefit the fetus and newborn, because pregnancy is a state of heightened metabolic activity, and the placenta is a key source of free radicals of oxygen and nitrogen. The multiple benefits of supplementary creatine arise from the fact that the creatine-phosphocreatine [PCr] system has physiologically important roles that include maintenance of intracellular ATP and acid–base balance, post-ischaemic recovery of protein synthesis, cerebral vasodilation, antioxidant actions, and stabilisation of lipid membranes. In the brain, creatine not only reduces lipid peroxidation and improves cerebral perfusion, its interaction with the benzodiazepine site of the GABA_A_ receptor is likely to counteract the effects of glutamate excitotoxicity – actions that may protect the preterm and term fetal brain from the effects of birth hypoxia. In this review we discuss the development of creatine synthesis during fetal life, the transfer of creatine from mother to fetus, and propose that creatine supplementation during pregnancy may have benefits for the fetus and neonate whenever oxidative stress or feto-placental hypoxia arise, as in cases of fetal growth restriction, premature birth, or when parturition is delayed or complicated by oxygen deprivation of the newborn.

## Introduction

### The need for a therapy that reduces the probability of perinatal morbidity and mortality

Two areas in obstetric and neonatal medicine that lack effective prophylactic treatments are premature birth and neonatal hypoxic-ischemic encephalopathy (HIE). It is well known that morbidity and mortality are higher for babies born pre-term [1]. Infants surviving premature birth can be left with severe, life-long disabilities of the nervous system such as cerebral palsy (CP), occurring not only as a result of the prematurity, but also from co-existing obstetric problems such as intrauterine infection, chronic fetal hypoxia, or with the problems that arise during resuscitation of the infant [2,3].

Systematic reviews show that maternal administration of corticosteroids for impending preterm birth significantly reduce the risk of neonatal death, respiratory distress, cerebroventricular haemorrhage, and necrotising enterocolitis, and clearly reduces the requirement for neonatal respiratory support and intensive care [4]. Antenatal magnesium sulfate administration has also been shown to reduce the risk of cerebral palsy when administered to women immediately prior to preterm birth [5]. Maternal administration of the xanthine oxidase inhibitor allopurinol is under trial as a means of protecting the fetal brain from hypoxia-induced oxidative stress [6]. However, these treatments are given late, and only when preterm labour is obvious and inevitable or the fetus is already clearly subjected to severe hypoxia. These treatments require tertiary level medical care, restricting their use to places with high levels of obstetric surveillance. In the case of allopurinol, significant concerns have been raised about its likely interference with normative and hypoxic regulation of the fetal circulation [7]. Notwithstanding the use of antenatal steroids and magnesium sulphate to lower the risk of hypoxia-induced brain injury at birth (pre-term or term), there are currently no accepted treatments that are recommended for use during the 2^nd^ and 3^rd^ trimester of pregnancy for the purpose of preventing birth-related hypoxic-ischemic encephalopathy.

Most perinatal demise still occurs in 3^rd^ world settings [8]. The World Health Organization estimates that up to 9 million newborn babies suffer birth hypoxia each year, leading to an estimated 1.2 million deaths (29% of global neonatal deaths), and similar numbers of infants who develop severe disability [8,9]. In addition to CP, cognitive and behavioural dysfunctions and psychiatric illnesses such as autism and schizophrenia are all disorders that occur more often in infants born from pregnancies with a history of obstetric complications, including prematurity, fetal growth restriction, or birth hypoxia [10]. The great challenge in developing an effective prophylactic treatment for preterm birth, birth-related HIE – or instances where these separate risks to the developing brain may combine – remains the inherent difficulty of predicting their occurrence [11]. Therefore, we need strategies that *prevent*, rather than rescue perinatal brain injury.

The most successful and commonly used clinical treatment for HIE is head cooling or total body cooling [11-13], but limitations include the need to apply it early after the onset of HIE. When applied in the 6 h immediately after birth, hypothermia does reduce mortality, and it significantly reduces the incidence of moderate to severe neurodevelopmental disability [13,14]. A recent multi-centre review concluded that no additional adverse effects or organ dysfunction arise from hypothermia treatment, but adverse outcomes have been reported when hypothermia is applied to healthy term infants [11]. Much of the delay in applying this rescue treatment – and therefore, in reducing its effectiveness - comes from the time taken to assess the neonate as being ‘suitable for treatment’.

Given the difficulty in: (i) the early prediction of preterm birth and perinatal hypoxia; (ii) the narrow therapeutic window for treatment: and (iii) the multi-organ dysfunction that is often an outcome - it is evident that the clinical practice of obstetrics still lacks effective means to prevent the morbidity that remains associated with preterm birth. Because neonatal hypothermia requires specialised equipment and trained personnel to administer it, its use and effectiveness in reducing the *global* burden of birth hypoxia is likely to remain limited.

## Review

### Creatine as a therapy for the 3^rd^ trimester

**Figure 1:**
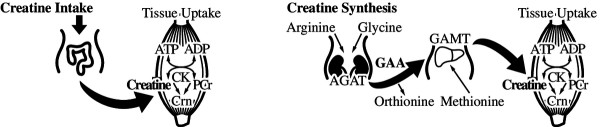
**Creatine is an amino acid derivative involved with cellular energy production.** In the form of energetically charged phosphocreatine (PCr), its primary function is to provide the phosphate group for regeneration of ATP from ADP in tissues of high and fluctuating energy demands. Human adults obtain approximately half of their daily requirement for creatine from a diet containing fresh fish, meat, and other animal products. The remainder is synthesized endogenously from arginine, glycine and methionine (methyl donor for GAMT reaction). This is a two-step process involving arginine:glycine aminotransferase (AGAT), principally in the kidney, producing guanidinoacetate (GAA), followed by hepatic guanidinoacetate methyltransferase (GAMT) activity producing creatine. Once synthesized, creatine is released from the liver into the circulation and taken up by most tissues, particularly muscle, by means of the creatine transporter. Inside the tissues a proportion of the creatine is phosphorylated to PCr, via the action of creatine kinase (CK). One important aspect of creatine biosynthesis is that the daily utilization of methyl groups on the GAMT reaction roughly equals the total daily intake of ‘labile’ methyl groups (methionine + choline) in an average diet. Thus, if methionine and choline levels are reduced, then endogenous creatine biosynthesis, responsible for half of our daily requirement for creatine, may be critically reduced.

The synthesis of creatine and its availability in a diet containing meat, milk products and fish is shown in Figure 1. Human adults obtain approximately half of their daily requirement for creatine from a diet containing meat, fish and other animal products, the remainder being synthesized endogenously from arginine, glycine and methionine in a two-step process involving arginine:glycine aminotransferase (AGAT), principally in the kidney, followed by hepatic methylation via guanidinoacetate methyltransferase (GAMT). Creatine is then released from the liver into the circulation and taken up by most tissues, especially muscle, by means of the creatine transporter protein (CrT). Once inside the tissues a proportion of the creatine is phosphorylated to form phosphocreatine via the action of creatine phosphokinase. Despite this endogenous synthesis, ingesting creatine increases the creatine content of skeletal muscle [15] and brain [16] indicating that in healthy people the intracellular pool of creatine is not fully saturated.

Creatine is an amino acid derivative involved with cellular energy production, but other beneficial effects of creatine have been identified, including antioxidant actions, stabilization of lipid membranes, and interactions with glutamate and GABA_A_ receptors that diminish excitotoxicity [17]. Recent animal experiments demonstrate that when given as a supplement to the mother’s diet during pregnancy, creatine protects the fetal brain, diaphragm, and kidney against hypoxic insult at term [18-20]. Creatine is not yet used in human pregnancy, but the positive results from these animal experiments suggest that creatine supplementation in the 2^nd^ or 3^rd^ trimester of human pregnancy could provide benefit to *all* pregnant women against the risk of antenatally or perinatally acquired brain injury, in much the same way that folate is now used to prevent neural tube defects in early pregnancy.

Creatine therapy can also be distinguished from hypothermia and magnesium sulphate in providing protection to a number of major organs against the physiological challenge of the transition from fetal to newborn life. We propose that the ‘pleiotropic’ properties of creatine will have benefits for many fetal tissues where vasoconstriction, oxidative stress, or glutamate toxicity may arise, in addition to its main function of maintaining mitochondrial function and ATP turnover.

Pregnancy is a state of heightened metabolic activity with extra nutritional requirements by the mother, and even in healthy pregnancy there is increased generation of oxygen and nitrogen free radicals [21], a key source of which is the placenta [22]. Fetal tissues, and the developing brain in particular, are vulnerable to oxidative stress especially if infection is present or inflammatory processes are otherwise triggered. The idea that anti-oxidants may protect the fetus against oxidative stress, especially in late pregnancy, has recently been reviewed [23]. The possibility that many problems in pregnancy arise from inadequate *nutrient* supply to the fetus, usually discussed mainly in relation to placenta transfer of glucose and essential amino acids, is now readily accepted [24]. However, in these cases, and especially in instances of fetal growth restriction, the fetal acquisition of creatine has never been examined.

A further reason for considering the use of creatine supplementation in pregnancy arises from the possibility that the preterm infant may become creatine depleted because it has not yet developed full capacity to synthesize creatine, or is unable to fully retain it by renal reabsorption. We must presume that the fetal source of creatine is placental transfer of maternal creatine until such time that the reno-hepatic capacity is sufficiently developed, but just when this occurs in human pregnancy is not yet known. In a recent study of preterm (32–35 weeks) and very preterm (28–29 weeks) babies, Lage et al. [25] showed that guanodinoacetic acid (GAA, the immediate precursor for creatine synthesis) concentrations in urine increased over the 35 (very preterm group) or 14 postnatal days (preterm group) until discharge, while urine creatine concentrations fell significantly. These results were interpreted as indicating that the accumulation of GAA was due to deficient hepatic conversion to creatine, and potential creatine deficiency was shown by the fall of urine creatine. Little is known about intestinal transport of creatine in preterm or term infants, but even if the baby was receiving nutrition by mouth, neither breast milk or infant formula contain significant amounts of creatine [26]. A further advantage of creatine loading during pregnancy for the preterm infant is that high levels of plasma creatine suppress the expression and activity of the enzyme arginine-glycine aminotransferase (AGAT) in the kidney, which would therefore have the effect of decreasing plasma levels of GAA. High levels of GAA in blood are known to be neurotoxic - the clinical manifestations of deficiency of the hepatic enzyme guanidine acetate methyltransferase (GAMT) and high circulating levels of GAA are intellectual disability, extrapyramidal movement disorders, and epilepsy [27,28].

There is a physiological cost of being born before the major organ systems have developed sufficiently to meet the demands of postnatal life; hence, the creatine status of prematurely born infants needs close attention. For human births before 34 weeks the heart, lung, kidney, and brain are immature to the extent that respiratory and cardiovascular support is often required, and resuscitation procedures often place the brain at further risk of hypoxic-ischemic injury [29,30]. Respiratory insufficiency also increases the demand on the neonatal diaphragm, a muscle that has not reached full functional maturity in preterm infants and is prone to fatigue [31]. Renal function may also be limited, as shown by a reduced number of glomerular generations in the kidneys of preterm infants [32]. Cardiovascular instability often results in highly variable perfusion of the brain that, due to immaturity of autoregulatory mechanisms, can result in cerebral haemorrhage [33]. Even late preterm infants, born 32–36 weeks gestation and who represent 80-85% of all preterm births, are at risk of respiratory problems, may require inotropic treatment, and suffer greater incidences of cerebral palsy and cognitive impairments. For infants born before 32 weeks, the incidence and severity of these problems is worse [34].

The creatine status of preterm infants is not known and should be the focus of further study. Some creatine reference values reported for human infants (326 μmol/l) and children (149 μmol/l) should be regarded as misleading because they were obtained from dried blood spots and are thereby ‘contaminated’ by the high creatine content of red blood cells [35]. Total creatine content of the brain (relative to all other metabolites, or calculated water content, identified by magnetic resonance spectroscopy) does not appear to change during *in utero* brain development [36] or, if it does, by not more than 1.25-fold between 22 and 39 weeks gestation [37,38]. However, with impaired brain development such as ventriculomegaly, total creatine content appears to be raised [36], which might be the consequence of sustained damage that leads to white matter gliosis [39]. Nevertheless, it is clear that acute hypoxic injury leads to depletion of ATP and phosphocreatine in human infants and children [40,41], confirming the animal studies and confirming that creatine supplementation should be investigated as a therapy to decrease the frequency and burden of hypoxic-ischaemic brain damage at birth.

### Creatine is a clinically important, safe nutritional supplement

Increasing the cellular pool of creatine/phosphocreatine through dietary creatine supplementation or subcutaneous injections has been shown to be neuroprotective in several animal models of neurodegenerative disorders and acquired central nervous system injury [42]. Clinical trials have shown that long-term creatine supplementation is well tolerated, slowing the accumulation of glutamate in the brain of early-onset Huntington’s Disease [43], without serious side effects when given over years in patients with Parkinson’s Disease [44], and improving short-term and long-term outcomes for children recovering from traumatic brain injury [45,46]. These neuroprotective functions of creatine have recently been extended by demonstrations that creatine improves cognitive function in normal and elderly people [47,48], and improves cognitive and motor skills in sleep-deprived subjects [49]. Consumption of creatine has also been shown to improve glucose tolerance in healthy sedentary males [50], and to improve muscle performance in elderly men and post-menopausal women [51]. Based on its actions on osteoblasts *in vitro*, creatine has been recommended as a treatment for osteoporosis in women [52]. Thus, the long-term use of creatine is being considered as a treatment to alleviate health problems ranging from type-2 diabetes, metabolic syndrome, sarcopenia, osteopenia, and cognitive decline.

Creatine, creatine monohydrate and creatine phosphate are all currently unclassified by the relevant statutory authorities in the EU (European Medicines Agency), UK (Medicine & Healthcare Products Regulatory Agency), USA (Federal Drug Administration) and Australia (Therapeutic Goods Administration), and have no assigned category for administration in pregnancy. In Australia, the Complementary Medicines Evaluation Committee’s (CMEC) most recent appraisal of creatine recommended that “there is insufficient evidence, at this time, of adverse consequences associated with the use of creatine and its salts to require any restrictions on dosage”. While the long-term use of creatine in adult humans has been considered in depth and appears to be safe [53-57], caution should nevertheless be exercised in recommending its use in human pregnancy as we still await detailed studies. In human infants, one study examined the oral administration of creatine to treat apnoea of prematurity [58]. Although the 14 day creatine treatment did not reduce the incidence of apnoea, it was well tolerated by the premature infants with no side effects noted.

The overwhelming evidence for considering creatine supplementation to be safe was recently summarised by Gualano et al. [57]. In our animal studies we have found no evidence of changes to maternal physiology that would raise concerns about recommending the use of creatine in human pregnancy. Nevertheless, given that pregnancy is normally associated with changes in fluid balance in women, consideration should be given to determining if creatine supplementation aggravates fluid shifts during pregnancy. Creatine is an osmolyte, and in very high concentrations could be associated with increased water uptake by cells. In non-pregnant human adults there has been some concern over possible deleterious effects of long-term, high-dose creatine supplementation on kidney function [59], but this was not confirmed by more recent work using ^51^Cr-EDTA clearance in normal patients and those with type-2 diabetes [60]. Studies measuring urine creatinine as a marker of kidney function should be interpreted with caution given that creatinine is the breakdown product of creatine and creatine phosphate. Thus, the presence of high urine creatinine would be expected during periods of high creatine consumption, and is not necessarily indicative of kidney damage [60].

### Why creatine?

Understanding how supplemental creatine works to prevent brain injury in the adult is useful in appreciating how it might be applied to prevent or attenuate fetal and neonatal brain injury. Many adult neuropathologies in which creatine has been shown to be beneficial also encompass the primary mechanisms of injury induced by hypoxia-ischemia (HI) in the immature brain, including mitochondrial dysfunction, impaired energy metabolism, excitotoxic injury and oxidative stress [42,61]. But it is the prospect that supplementary creatine can act as *a multi-organ protectant* for the fetus and neonate that gives it a promise not provided by the current therapies; e.g., magnesium sulphate or, in particular, head cooling.

The ‘pleiotrophic’ properties of creatine [62] arise from the fact that in addition to acting as the essential *spatio-temporal* metabolite by maintaining ATP via transfer of the phosphoryl group to ADP, the creatine/phosphocreatine (PCr) system has a number of other physiologically important roles:

***(i), acid–base balance:*** the rephosphorylation of ADP utilizes a proton (PCr + Mg ADP + H^+^ → creatine + MgATP), thus reducing the acidity of the intracellular environment under hypoxic conditions [62,63]. This may be important in maintaining muscle contractile function [64,65]. However, while it has been thought that the minimization of acid–base changes during hypoxia in the presence of creatine were due only to changes in cellular energy dynamics (i.e. donation of proton with PCr), the study of Lawler et al. show that creatine, when present at high concentrations in the cell, and unlike PCr, has an inherent ability to scavenge free radicals [66]. Thus the creatine/phosphocreatine system has the ability to modulate changes of intracellular acid–base balance that might arise during periods of severe hypoxia in a direct, antioxidant manner.

***(ii), antioxidant actions:*** Perhaps related to this effect on H + accumulation, creatine has been shown to be a mild antioxidant in adult rat gastrocnemius *in vitro*[66]. Furthermore, our results show the increased level of malondialdehyde (product of lipid peroxidation) caused by intrapartum hypoxia in spiny mouse pup brain is completely prevented by maternal creatine supplementation from mid-pregnancy [20]. Creatine supplementation reduces the increase of 8-hydroxydeoxyguanosine, a biomarker of oxidative damage to DNA, which occurs as part of the natural aging process [53,67]. Creatine has the property of quenching superoxide, as opposed to increasing the expression of antioxidant enzymes or attenuating ROS production [68,69].

***(iii), post-ischemic recovery of protein synthesis:*** recovery of the decreased protein synthesis that precedes neuronal cell loss in the post-ischemic brain is faster after creatine pre-treatment in the oxygen-glucose-deprived adult rat [70] and fetal guinea pig hippocampal slices [71], resulting in a more favourable histological outcome. In muscle, post-ischemic reactive oxygen species (ROS) production has been shown to disturb Ca^2+^ homeostasis and increase intracellular Ca^2+^ levels resulting in a rise in protease activity, thus increasing contractile protein degradation [72]. Creatine has been shown to attenuate these effects by aiding in the maintenance of ionic balance and by promoting muscle protein synthesis.

***(iv), improved cerebral vascular function:*** mice pre-treated with creatine showed faster recovery of cerebral blood flow during reperfusion after middle cerebral artery occlusion, possibly due to greater dilator responses to extra-luminal potassium and acidosis [73].

***(v), interaction with the benzodiazepine receptor:*** specific binding of creatine to the benzodiazepine binding site of the GABA_A_ receptor has been shown in the chick brain [74], and animals fed a creatine supplemented diet showed increased GABAergic activity in some brain regions (striatum) [75]. The anti-excitatory effect of increased GABA_A_ receptor activity is likely to be protective for the immature brain (reviewed by [76]).

***(vi), promoting the uptake of glutamate:*** uptake of glutamate into synaptic vesicles is an ATP-dependent process, thereby depending on creatine to maintain ATP [77]. PCr has also been shown to promote the uptake of glutamate into synaptic vesicles [78], and may account for the neuroprotective capacity of creatine against glutamate toxicity in neuronal cell culture [79].

***(vii), stabilization of lipid membranes:*** PCr interacts with phospholipid membranes to stabilize membranes and prevent membrane permeabilisation [80].

### Evidence that creatine protects against HIE at birth

Hypoxia-ischemia at birth occurs in approximately 4 per 1000 live term births [81], and depending on its severity and duration 4-8% of these infants will die. Those infants that do survive experience severe health problems, stemming from irreversible multi-organ damage to the brain, kidneys, heart and lungs [82]. The brain has received the greatest attention with respect to hypoxia-ischemia (HI) given that 20-70% of survivors have brain damage with lifelong effects including mental and physical disability, cerebral palsy and seizures [2,81]. However, the recognition and treatment of other systemic complications of HI, including acute kidney injury, muscle damage, and compromise of heart function are essential for overall homeostasis and thus survival.

Experimental studies in the precocial spiny mouse show that a diet enriched with 5% creatine (approx. 1.36 g/kg body mass/day) given from 0.5 gestation result in a 10-30% increase in creatine content in fetal tissues (incl. heart, kidney, liver, brain and muscle) and a 2-fold increase in the placenta at term [83]. Fetal and maternal liver creatine content was increased much more than in muscle; similar findings of greatly increased hepatic creatine after oral supplementation have been made in non-pregnant animals [84]. The increased content of creatine in the fetal liver at term may act as an additional creatine pool available to the neonate that may be, for the reasons given above, at risk of creatine depletion. Importantly, the exposure to increased creatine did not alter the protein expression of enzymes in the neonatal kidney and liver - AGAT, GAMT respectively - required for creatine synthesis postnatally [85]. Furthermore, this creatine treatment did not have any obvious effects on maternal health status or body composition (Ellery et al., unpublished data). These findings suggest no harmful effects of the maternal creatine treatment on the mother or neonate. While creatine supplementation had no effect on fetal weight in normal pregnancies, effects where fetal growth restriction is present are yet to be determined. Our studies showing the protective effects of this creatine treatment on brain structure [20], postnatal behaviour [83], diaphragm [18], and kidney structure and function [19] following asphyxial birth in the spiny mouse gives reason to think that creatine may also benefit the preterm infants in their premature transition from fetal to newborn life.

Creatine and phosphocreatine (PCr) act by maintaining intracellular ATP and thereby enable cells to prolong mitochondrial function and resist the initial metabolic collapse following HI [86]. Creatine may also target some of the secondary responses to HI, including reducing oxidative stress and promoting the post-ischemic recovery of protein synthesis [71]. However, effective resistance to the initial phase of a hypoxic challenge may prevent occurrence of the secondary wave of cell death altogether.

A limitation encountered in adult studies of the neuroprotective properties of creatine is the slow transfer of exogenous creatine into the brain, thought to be due to the creatine transporter at the blood brain barrier working close to saturation, and leading to the view that long-term, high dose creatine administration is required to significantly increase the content of creatine in the brain [87,88]. Indeed, early studies in mice [89], and humans [16] appeared to show that pre-treatment with creatine for several weeks to a month or more were required to raise brain creatine levels and to provide for therapeutic benefits against cerebral ischaemia [89]. However, shorter supplement protocols have shown that creatine levels in the brain can be increased. Lyoo et al. [90] demonstrated significantly increased human brain creatine levels using a supplementation protocol of 0.3 g/kg/day for 7 days, followed by another seven days of 0.03 g/kg/day. This result was consistent with Pan and Takahashi [91] who reported significantly increased creatine concentrations in the human brain following creatine supplementation at 20 g/day for 7 days. As for the longer supplementation protocol described by Dechent et al. [16], the studies using shorter supplement protocols used ^1^H-MRS and phosphorus magnetic resonance spectroscopy (^31^P-MRS) to determine brain total creatine concentrations. Both techniques enabled the correlation of the change in brain creatine with changes in high-energy phosphate metabolism, and despite differences in the creatine supplementation protocols and methods used to measure creatine concentrations, significant increases in brain creatine concentrations were observed in all cases.

However, creatine does appear to enter the immature brain more readily, possibly because of higher expression of the creatine transporter in endothelial cells of the choroid plexus [92], allowing the developing brain to utilize peripheral creatine more so than in the mature brain (reviewed by [93]). In the postnatal rat brain, a significant increase in the PCr/NTP (nucleoside triphosphate) ratio could be achieved by subcutaneous injections of creatine at day 10, but not at day 20, and this also significantly increased the recovery of cerebral PCr/NTP ratio within 2 h post-hypoxia [86]. Creatine pre-treatment also reduced brain oedema, and the incidence of severe cystic cerebral infarction following hypoxic-ischaemia in 7 day-old rats [71]. The creatine transporter is widely expressed in the fetal rat brain, suggesting that the immature brain may have a greater capacity to take up creatine from the circulation compared to the more mature brain, although evidence also suggests that creatine may enter the immature brain more easily by diffusion - i.e., a non-carrier pathway [93]. Hence, it must be considered that creatine supplementation given to pregnant women even for only several weeks in late pregnancy is likely to increase the resistance of the fetal brain to oxygen deprivation or acidemic/hypercapnia at birth, or if poor ventilatory efforts by the neonate require resuscitative procedures immediately after birth.

### What do we know of creatine metabolism in pregnancy?

Surprisingly little is known about the creatine status of pregnant women, particularly that subset of women who avoid meat and dairy products (vegetarians, vegans) and therefore rely entirely on their own capacity to synthesize creatine. In adult males, the creatine content of muscles is ~30% lower in vegetarians compared to those that eat meat, fish and dairy [94]. Notwithstanding the greater exposure to phytoestrogens and the nearly 5 fold greater risk of hypospadias in boys [95], a vegetarian or vegan diet does not appear to be associated with increased obstetric problems or adverse pregnancy outcomes, although this is usually discussed only in terms of iron, vitamin B12 and total protein, and not creatine [96]. The possibility that the amount of creatine available for transfer to the feto-placental unit is limited by this dietary choice in pregnancy should be examined more closely.

In humans, and probably most omnivore species, creatine is actively transported across the placenta; indeed it appears to first accumulate in the placenta and then diffuse down a concentration gradient into the fetal circulation [97-99]. In animal studies, supplementary creatine given in the diet from mid-pregnancy, results in a 2-fold increase in the placental creatine concentration [83]. In the human placenta, the mRNA for the creatine transporter (CrT) is detected early in pregnancy [100], and the capacity for maternal-fetal transfer of creatine is present from at least 13 weeks of gestation [101]. CrT protein expression is present in the syncytiotrophoblast of the term human placenta (unpublished data), consistent with its placement for uptake of creatine from the maternal circulation, but the mechanism(s) for its release into the fetal circulation is still a matter of conjecture. It has not yet been satisfactorily established in any species if the placenta expresses the creatine synthesising enzymes AGAT and GAMT and is itself able to synthesise and release creatine.

As a metabolically active organ, it is likely that the placenta itself has a creatine requirement - expression of several creatine kinase isoforms, which are tightly coupled with cellular energy requirements, has been shown to peak in the term placenta [102]. Creatine kinase expression also increases in the rat placenta during pregnancy, when its decrease near to term is thought to signal placental senescence [103], but whether this also occurs in the human placenta is not known. The increase of placental creatine kinase expression may follow from increased steroidogenesis, solute transport, and other placental activities such as glucose-glycogen storage and amino acid metabolism, which are all energy-driven processes that increase towards term.

Observations on creatine kinase expression in fetal tissues during the last trimester of pregnancy are understandably few, but on the assumption that increased creatine kinase expression follows the need for increased production of high-energy phosphates, it would not necessarily follow that creatine kinase expression in fetal tissues would match the changes that occur in the placenta. On the other hand, they might, if indeed creatine kinase expression is modulated by oestrogen – and in particular, ubiquitous mitochondrial [uMt] CK - as has been suggested for placental creatine kinase [103]. Thus, total CK and mitochondrial CK expression increased with gestational age in skeletal muscle samples obtained from preterm infants 28 to 36 weeks gestation [104], (although these samples were obtained at post-mortem and at times up to 6 days after birth, and confounding factors must include the babies condition leading to their early death and postnatal muscle activity). Increased myocardial CK expression in late gestation fetal sheep can be explained by increased demands on the heart with accelerating fetal growth [105], but increased muscle ‘work’ would be a less likely explanation for the increased CK expression in fetal peripheral muscles.

In healthy women muscle creatine content increases from [pre-pregnant] 200 mmol/kg alkali-soluble protein (ASP) to 223 mmol/ kg ASP at 18 weeks and 233 mmol/ kg ASP at 36 weeks gestation [106]. This muscle creatine loading may to be necessary to meet the uterine energy demands of labour, which requires prolonged, recurrent episodes of very intense uterine smooth muscle contractions [106]. The impact of creatine supplementation on the pregnant uterus bears consideration, but except for a small study on the effects of creatine on structure of the rat myometrium [106], the involvement of creatine in myometrial activity, and the increased activity associated with labour, has been little studied. It has been suggested that decreased uterine contractility following prolonged contractions might be protective in preventing hypoxic damage to the uterus - and therefore, progressive fetal hypoxia [107]. However, this physiological response to uterine ischemia might nevertheless have the consequence of causing progressive failure of uterine ‘effort’ at term (i.e., uterine dystonia), a situation that could be compensated for by creatine supplementation in late pregnancy.

### Creatine synthesis in the fetus and newborn

While it is likely that the ‘dietary’ source of creatine for the fetus is the maternal circulation, it is presently unclear when the capacity for creatine synthesis develops in the fetus. Studies in the precocial spiny mouse suggest that the reno-hepatic axis of creatine synthesis develops late in gestation [108], and if this applies to human pregnancy would imply that infants born prematurely have an under-developed capacity for creatine synthesis and might therefore be at risk of becoming creatine deficient. The dependence of the fetus on maternal creatine is suggested by the fact that those rare infants born without the capacity for creatine synthesis develop signs of neurological dysfunction in the days and weeks *after* birth [109,110] suggesting that they were not creatine deficient before this, even though they are unable to produce creatine of their own. Important also for the neonate is its capacity to reabsorb creatine from glomerular filtrate. CrT protein is highly expressed in the proximal tubule of the adult kidney and creatine excretion is very low [111]. As discussed above, whether this is so for the preterm infant is not yet fully known, and urinary creatine loss may be important because preterm birth is often associated with impaired renal function [112]. Urine composition has been used in the assessment of the risk of developing HIE in neonates (e.g., urinary lactate/creatinine ratio) [113], but the ability of the kidney to retain creatine in preterm infants has not yet been examined.

### Creatine, a multi-organ protectant against hypoxic injury in the neonate

Our studies in the spiny mouse have shown that maternal dietary creatine supplementation prevents hypoxic injury to multiple organs including the brain [20], diaphragm [18], limb skeletal muscle (unpublished data) and kidney [19] at birth, increasing survival of the hypoxic term fetus, and also improving postnatal growth [83]. Acute kidney injury (AKI) and subsequent neonatal renal failure are significant complications of HI; approximately 50% of asphyxiated neonates present with some form of AKI, and the severity of the condition strongly correlates with subsequent morbidity, mortality, and poor neurological outcomes [114]. The injury to the kidneys stems not only from oxygen deprivation and ischemic tissue damage, but also from the increased workload imposed on the kidney to amend the changes to acid–base and electrolyte levels which occur with the global insult of hypoxia [115]. Our recent work on the kidney has shown that the structural integrity of the kidney is significantly impaired 24 h after birth hypoxia, with evidence of shrunken glomeruli, dilated tubules, and impaired migration of the tubules into the medulla [19]. Supporting our observation of tubular injury, the expression of neutrophil gelatinase-associated lipocalin (*Ngal*), a marker of tubular injury, is significantly up-regulated after birth hypoxia. These changes in kidney structure, and markers of injury are completely prevented by maternal dietary creatine supplementation.

### Which obstetric population might benefit from creatine supplementation?

The potential roles of creatine may be relevant to an array of pregnancy conditions leading to preterm birth, and therefore to high risk of HIE. One such condition is preeclampsia, a common syndrome of pregnancy that manifests after 20 weeks gestation with new-onset hypertension alongside maternal end-organ dysfunction and/or intrauterine fetal growth restriction [116], and is a factor associated with nearly 50% of preterm births [117]. It affects around 5% of all pregnancies [118], and is characterized by significantly elevated levels of oxidative stress within the maternal-fetal unit [119]. Gestational diabetes mellitus is another common disease of human pregnancy also affecting around 7% of women and characterized by a state of increased oxidative stress and increased risk of preterm birth [120,121]. These pregnancy conditions along with many others warrant investigation as to whether creatine may afford some protective or therapeutic benefit.

In addition to pre-eclampsia, other obstetric conditions that must be managed conservatively include cervical incompetence, preterm premature rupture of the membranes (PPROM), partial placental abruption and placenta praevia, and fetal growth restriction. Fetal hypoxia, hypoglycaemia and activation of the fetal hypothalamic-adrenal axis arise as consequences in many of these conditions, leading not only to fetal growth restriction and preterm delivery but also, potentially, to fetal death or stillbirth [122,123]. Intrauterine demise of the fetus will always involve mitochondrial energy failure. Therapeutic interventions that include bed rest [124], low dose aspirin [125], or conventional nutrient supplementation [126] have been shown to have limited success in reducing perinatal morbidity and mortality. Creatine supplementation of the maternal diet is consistent with conservative management and would not interfere with the increased surveillance of such high-risk patients that is now standard clinical practice. The absence of any clinical trial at the present time of creatine supplementation in human pregnancy is an obvious barrier to the implementation of this treatment, although a trial of creatine for neuroprotection of the human fetus at term has recently been proposed [127]. Uterine smooth muscle contains remarkably low levels of creatine, and ATP and phosphocreatine are readily depleted by uterine activity [128]. Given that uterine activity in late gestation can be uncoordinated and labour sometimes ‘fail to progress’, a creatine supplementation program in pregnant women might also decrease the caesarean section rate.

## Conclusion

Creatine is considered a safe nutritional supplement for adult humans and has been shown not only to increase muscle mass and performance, prevent disease-induced muscle atrophy and improve rehabilitation, but also to strengthen cellular energetics in general [129]. By maintaining tissue energy levels and preventing oxidative stress, elevating tissue creatine levels by dietary supplementation is able to prevent tissue injury induced by hypoxia and circulatory collapse. In addition to yielding ATP, the dephosphorylation of creatine utilizes free protons and ADP, thereby reducing the fall of intracellular pH and aiding in the stabilization of the mitochondrial membrane potential. Creatine may also have important modulatory effects on the glutamate and GABA-A receptor systems that raise the threshold for the onset of excitotoxicity in the brain. The importance of creatine may extend beyond protecting the brain to preventing damage to other organs. In pregnancy, hypoxia, inflammation, and oxidative stress are reasonably common events that lead to compromise of not only the brain, but other important organ systems, rendering them particularly vulnerable to hypoxic-ischemic damage that can occur at birth, particularly preterm birth. The use of creatine in human pregnancy and neonatal practice should therefore be evaluated as a possible prophylactic therapy in its own right, or as an adjunct to conventional treatments such as magnesium sulphate when preterm labour is likely, or in HIE when hypothermia is used.

## Competing interests

There are no conflicts of interest. None of the listed authors received any payment for their participation in writing this manuscript.

## Authors’ contributions

DWW, HD, RJS, and ZI wrote the original draft; SE and DL contributed significantly to redrafting and to the final manuscript. All listed authors were involved in revising the draft and producing the final manuscript. All authors read and approved the final manuscript.

## Pre-publication history

The pre-publication history for this paper can be accessed here:

http://www.biomedcentral.com/1471-2393/14/150/prepub
